# Press Conduction Welding for Secondary Bonding of Aircraft Skin/Stiffener Assemblies Using Carbon Fiber/PEKK Thermoplastic Composites and PEI Adhesive

**DOI:** 10.3390/polym16060750

**Published:** 2024-03-09

**Authors:** Hyunseok Choi, Chan-Joo Lee, Yong-Jun Jeon, Woo-Chun Choi, Dongearn Kim

**Affiliations:** 1 Department of Mechanical Engineering, Korea University, Seoul 02841, Republic of Korea; hschoi01@korea.ac.kr; 2 Korea Institute of Industrial Technology (KITECH), Incheon 21999, Republic of Korea

**Keywords:** thermoplastic composites, conduction welding, carbon fiber/PEKK, secondary bonding, aerospace materials, PEI adhesive, bonding strength, composite manufacturing, press welding

## Abstract

This study investigates the secondary bonding of aircraft skin/stiffener assemblies using press conduction welding with carbon fiber/polyetherketoneketone thermoplastic composites and polyetherimide adhesive. Recognizing the challenges posed by conventional welding methods in maintaining material integrity and uniformity, this research explores an alternative methodology that mitigates these issues while ensuring high-strength bonds. The press conduction welding parameters were selected based on single-lap shear tests and applied in the bonding of skin and omega stiffener components. The temperature range was determined using differential scanning calorimetry. The pressure was held at 1 MPa for 180 s. The welding temperature that produced a high-bonding strength was identified experimentally; these key variables were then used in the welding process of the skin and omega stiffener. By analyzing how the fibers tear and the effectiveness of interdiffusion between the plies, we were able to gain insights into the bonding strength and fractured surface. The findings suggest that press conduction welding provides a viable route for secondary bonding in thermoplastic composite structures, highlighting its advantages in terms of processing efficiency and integrity. This study contributes to the understanding of the mechanical behaviors of bonded joints and underscores the significance of temperature control in the welding process.

## 1. Introduction

Carbon fiber-reinforced composites are increasingly recognized as structural materials across various applications owing to their superior specific strength, particularly in the aerospace sector [1,2]. However, aircraft are frequently exposed to impact loads from bird strikes and landing [3], and despite their notable specific strength, carbon fiber-reinforced polymers demonstrate inadequate resistance to impact [4,5]. To address this limitation, the development of high-performance composites utilizing thermoplastic polymer matrices with exceptional impact and damage resistance has been proposed. Recent advancements have focused on thermoplastic composites with polyetherketoneketone (PEKK) matrices noted for their low melting temperature, ease of processing, and remarkable mechanical properties [6,7,8]. PEKK-based thermoplastic composites offer high-impact strength and reduced consolidation cycle times and are associated with simple repair and welding processes owing to the reversible reactivity of the matrix. The repair and welding techniques for these composites encompass various heating methods, including conduction, induction, resistance, and ultrasonic. Early studies explored the application of resistance heating-based welding using a carbon fiber heating element to ensure integrity and avoid the introduction of foreign materials within the laminate [9,10,11]. However, owing to issues such as disruption of the reinforcement phase and reduced temperature uniformity—attributable to the uneven resistance of carbon fiber heating elements—research pivoted toward stainless steel mesh heating elements [12,13,14]. These alternative heating elements produce welded joints with superior strength and consistency compared with those produced using carbon fiber heating elements. Recent studies have also investigated the use of carbon nanotubes (CNTs) as heating elements [15,16,17] and have reported that welding carbon fiber/PEKK (CF/PEKK) laminates with a CNT web yielded a flexible structure with an impressive single-lap shear strength of approximately 30 MPa.

Induction heating welding utilizes the eddy currents and magnetic polarization generated in conductive materials when subjected to alternating-current electromagnetic fields [18]. Conductive materials, such as carbon fiber, metal mesh, and metal particles, serve as susceptors. Utilizing carbon fiber as a susceptor without an additional component results in the material adjacent to the coil heating first, necessitating effective cooling for uniform heat generation [19]. This induction heating approach has been applied in the continuous welding of large-scale components [20,21].

Conduction welding is a technique that requires minimal energy and has been extensively studied in various applications using robotic tools [22]. In a conduction welding experiment of CF/PEKK laminate, a single lap-shear test was performed by heating one side of the laminate to 400 °C, which resulted in complete melting. The test produced an average failure load of 18,841 N; the study used the finite element method to compare the results with actual experimental data and proposed insights to predict failure modes. The results showed that the ply at the interface and the surrounding ply must be taken into account in the welding process.

The bonding of most thermoplastic composites is achieved by heating to melt the matrix, followed by the contact and integration of the adherends. However, this method has several challenges, such as deformation, polymer matrix degradation, and fiber spreading in the heat-affected zone during the heating process. To mitigate these issues, temperature control or postprocessing techniques are typically employed. Secondary bonding presents a solution devoid of these complications. Specifically, amorphous interlaminar bonding involves the application of an amorphous adhesive to a semicrystalline thermoplastic matrix, which minimizes deformation while ensuring high bonding strength [23]. This method enables polymer interdiffusion at the interface upon heating above the glass transition temperature (*T*_g_) of the adhesive but below the melting temperature of the matrix. Consequently, the method can minimize deformation of the adherend and realize energy savings by using relatively low processing temperatures. The use of thermoplastic composite parts in aircraft primary structures has limited application. So far, they have only been applied using methods such as induction and resistance welding without being welded by secondary bonding. The PEI adhesive and PEKK matrix are great for these parts because they have excellent miscibility [24,25] and physical properties thanks to the characteristics of aromatic polymers. However, not enough research has been done on secondary bonding for aircraft parts. We will investigate temperature as a key variable to better understand the welding process. Given their size, traditional welding of aircraft structures usually involves autoclave or oven consolidation methods. A potential limitation of press conduction welding is that it requires large pressurization devices to weld large parts, resulting in high initial investment costs. However, press conduction welding offers a markedly shorter and more efficient processing time, along with benefits in terms of load transfer clarity and reduced internal laminate defects. Despite these advantages, research on the application of press conduction welding for secondary bonding remains limited.

**Figure 1:**
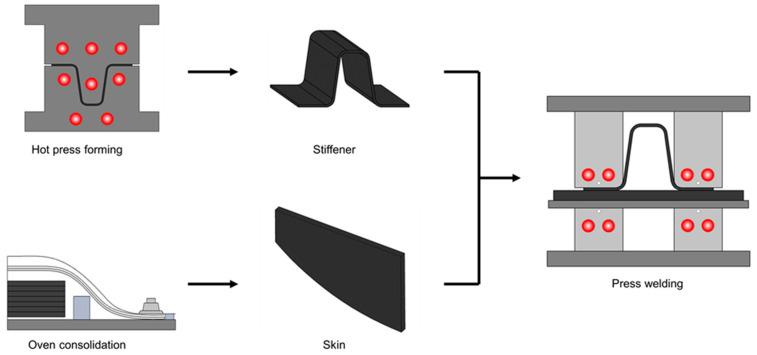
Schematic of secondary bonding of skin and omega stiffener by press conduction welding.

This study aims to explore the application of amorphous interlaminar bonding within the context of aircraft skin and stiffener structures with a focus on secondary bonding. Preliminary experiments were conducted to select parameters for the welding process based on single-lap shear tests. Utilizing the identified process parameters, skin and omega stiffeners were joined and analyzed. Figure 1 shows a schematic summarizing the research approach. The adherend was constructed from a CF/PEKK-based thermoplastic composite, employing a polyetherimide (PEI) film as the adhesive. The welding process employed press welding techniques utilizing conduction heating. The outcome of this demonstration was assessed in terms of bonding strength and process characteristics through pull-off testing.

## 2. Investigation of Welding Temperature Range Based on Single-Lap Shear Tests

### 2.1. Materials

The experiments utilized Solvay’s APC prepreg, which incorporates AS4D 12K carbon fibers within an aromatic polymer (PEKK) matrix. This prepreg has a resin content of 34 wt%. According to the manufacturer’s technical data sheet, the PEKK’s terephthaloyl to isophthaloyl ratio is 7:3, and the melting point of the neat resin is 337 °C. The *T*_g_ of PEKK is reported to be 159 °C, while the prepreg demonstrates a tensile strength of 102 MPa and a tensile modulus of 1.5 GPa. The unidirectional tape was arranged in a quasi-isotropic layup sequence of [+45/0/−45/90]_2s_, resulting in a consolidated ply thickness of 0.14 mm. For the welding process, a PEI adhesive film (Riyadh, Saudi Arabia, SABIC ULTEM™ Resin 1000) was applied, featuring a thickness of 0.175 mm and a *T*_g_ of 217 °C.

### 2.2. Single-Lap Shear Test

**Figure 2:**
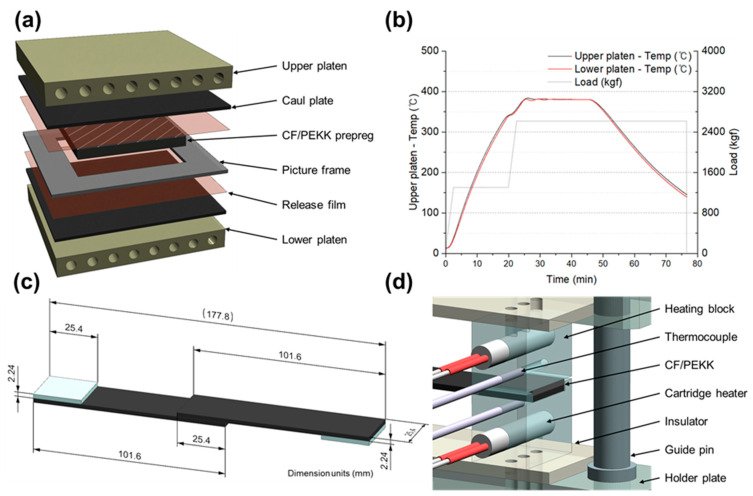
Single-lap shear test for establishing process parameters. (**a**) Hot press based on prepreg compression molding in carbon fiber (CF)/polyetherketoneketone (PEKK) laminate flat panel. (**b**) Consolidation cycle for thermoplastic composite flat panel. (**c**) Dimensions of the single-lap shear test specimen and tool configuration. (**d**) Schematic of single-lap welding jig.

Figure 2 summarizes the single-lap shear test utilized to determine the welding process parameters and to achieve laminate consolidation in the experiment. The laminate was fabricated by stacking 16 plies of CF/PEKK prepreg in a quasi-isotropic layup sequence. A schematic of the hot press platen and the components applied at this stage is depicted in Figure 2a. The hot press platen, made of Inconel, was chosen to minimize dimensional changes due to thermal expansion, while a graphite cowl plate was employed to ensure temperature uniformity. A cartridge heater was applied to heat the platen, and the cartridge heater was custom-made to minimize the temperature difference between the outer and central sides. The thermocouple was installed at the center (5 mm inside the surface of the platen) and was configured to respond flexibly to heating and cooling. The thermocouple diameter was 0.1 mm, which was selected considering durability and measuring rate. The picture frame, fabricated from mild steel (SS400), was designed to limit fiber spreading and resin compression. The thickness dimension was precisely machined to 2.24 mm, considering the consolidated ply thickness of the prepreg. The release film for demolding utilized a film (thickness = 75 μm) coated on one side of a polyimide film with polytetrafluoroethylene.

The temporal variations of temperature and load curves, critical for laminate consolidation, are shown in Figure 2b. The maximum dimensions of the tested specimens were set to 160 × 160 mm. The maximum temperature was 380 °C, identified as the consolidation reference temperature, and the maximum applied pressure was 1 MPa. The lamination process was divided into compression and heating stages to reduce control error. In the first stage, the pressure was fixed at 50% of the 1 MPa target pressure (i.e., 0.5 MPa), and the specimen was heated to 90% of the maximum temperature (i.e., 342 °C). The second stage was started upon reaching 342 °C. In this stage, the pressure was increased to 1 MPa, and the specimen was heated to 380 °C. Upon reaching 380 °C, a 15-min hold time was initiated before cooling at a rate of 17 °C/min. Throughout this stage, the pressure was maintained at 1 MPa; that is, the pressure was only removed after the cooling process concluded. Figure 2c illustrates the dimensions of the single-lap shear test specimen, adhering to the American Society for Testing and Materials D5868 standards [26].

For the bonding test of the fiber-reinforced composite material, the overlap length was set at 25.4 mm, and the length of each specimen was set at 101.6 mm. The bonding of the single-lap shear test specimen was performed using a specially fabricated jig, as shown in Figure 2d. This jig, designed to accommodate one specimen per cycle, ensures precise heating and pressure application. The jig’s heating system employed a proportional–integral–derivative (PID) control method with cartridge heaters. Insulation composed of glass fiber ceramic composite material was used on both the top and bottom to ensure temperature uniformity. Stability in thermal expansion was ensured by installing heating blocks made of NAK80 steel at the top and bottom, while guide pins on the holder plate facilitated the accurate positioning of the specimens. The specimen’s thickness and dimensions were designed to include a gap in the heater block, which acted as a stopper and a guide rail, thus allowing the specimen to endure additional compression of up to 0.3 mm.

**Figure 3:**
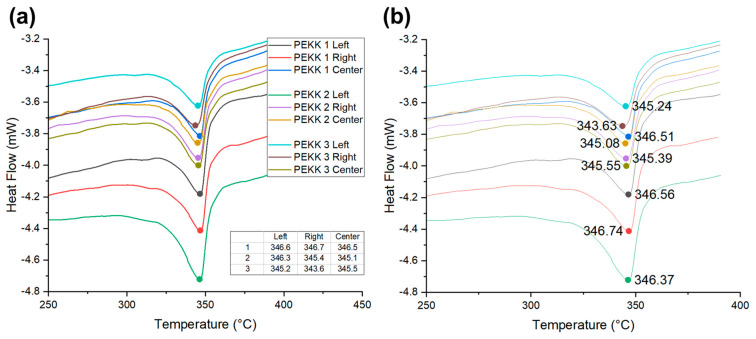
Differential scanning calorimetry data for welding temperature range: (**a**) PEKK laminate and (**b**) polyetherimide (PEI) adhesive film.

Figure 3 presents the results from differential scanning calorimetry (DSC) analysis aimed at identifying the optimal temperature range for the welding process. This analysis focused on the determination of the melting point of the PEKK laminate and the *T*_g_ of the PEI adhesive film. The experiments were performed using a TA-100 SDT Q600 instrument under nitrogen atmosphere conditions, with cooling facilitated air exposure at 24 °C, omitting the need for a separate cooling apparatus. Figure 3a reveals that the average melting point of the PEKK laminate was marginally above the manufacturer’s specified value with values equal to approximately 345 °C in the nine tested samples. This discrepancy is attributed to the thermal history effects arising from the secondary heating for lamination consolidation. To enhance crystallinity while consolidating the laminate, a cooling rate of 17 °C/min was applied, which is relatively low. The prepreg indicated relatively low results in comparison to the melting point of the laminate, caused by thermal history. Therefore, it is assumed that the melting point of materials that have undergone the laminate consolidation process has increased due to the dissociation of spherulite crystalline.

The *T*_g_ of the PEI adhesive film was approximately 216 °C, which aligns with the manufacturer’s specifications. The minimum temperature required for the process was determined to ensure the PEI adhesive film melts sufficiently. The DSC experiment results showed that PEI has a *T*_g_ of 216 °C with a clear inflection point. However, determining sufficient fluidity based on *T*_g_ is difficult, so a higher temperature of 260 °C was set as the lower limit of the process temperature, as shown in Figure 3b, where stable heating occurs after the endothermic reaction. Consequently, a processing temperature range was defined (from 260 °C (at which the PEI adhesive film attains adequate fluidity above its *T*_g_) to 340 °C (which approaches the melting temperature (*T*_m_) of the PEKK matrix)). The rationale behind this range was to avoid temperatures exceeding the *T*_m_ of PEKK, because the matrix phase becomes considerably fluid above the *T*_m_ of PEKK, challenging the maintenance of shape and leading to potential fiber spreading. To explore the effect of temperature on the process, welding tests were conducted at 20 °C intervals within this range. The welding process parameters for the single-lap shear test are listed in Table 1.

**Figure 4:**
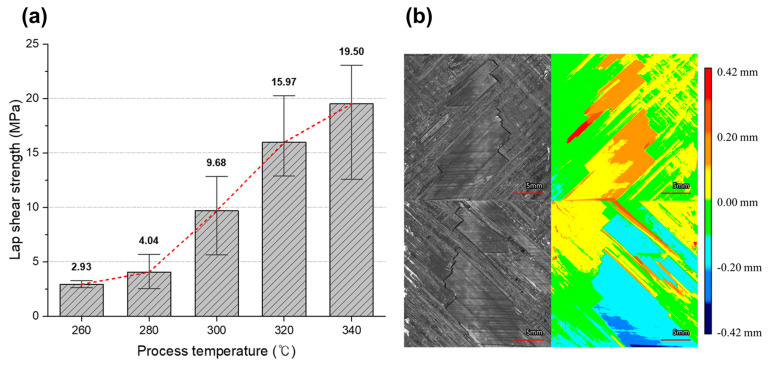
(**a**) Relationship between failure load and welding temperature in single-lap shear test. (**b**) Morphological analysis of fractured surface in the case of the welding temperature of 340 °C.

The single lap shear test results (according to welding temperature and morphological analysis of the fractured surface) are shown in Figure 4. Figure 4a shows the bar chart of the maximum failure load as a function of the welding temperature. Figure 4a shows a positive correlation between the welding temperature and bonding strength. During the tests, the samples welded at 260 and 280 °C frequently fractured under the clamping load, thus suggesting that the PEI adhesive film may not achieve sufficient fluidity at these temperatures. This was further indicated by the shape of the PEI film, which did not change after welding at these temperatures. However, at higher welding temperatures, the surface structure of the adherend was replicated on the remaining adhesive film, thus implying an increase in the fluidity of the adhesive film. This correlates with the observed enhancement in bonding strength. The morphology of the fractured surface of the sample welded at 340 °C was analyzed by a noncontact imaging method; the results are shown in Figure 4b. Fracture surface analysis was carried out at 340 °C, which represents the highest bond strength condition. The analysis showed that the ply of the counterpart remained on the adherend’s surface. In most areas, the ply was separated from the counterpart or was attached to the fracture surface of the adherend, resulting in a protruding form. This indicates a mixture of adhesive failure and fiber tear failure. It is believed that fiber tear failure occurred in the region where the PEI adhesive film interfused with the PEKK matrix.

The data show no significant bonding strength differences at 260 and 280 °C, but a marked improvement is evident at the temperatures of 300, 320, and 340 °C. This pattern suggests that the PEKK matrix begins to soften at approximately 300 °C with polymer interdiffusion increasing as the temperature approaches the melting point of approximately 345 °C. This behavior indicates that enhanced interdiffusion between the PEKK matrix and the PEI adhesive contributes to the observed increase in bonding strength. This hypothesis is supported by the analysis of the fracture surface in Figure 4b, where ply fractures within the PEKK matrix—attributed to interdiffusion with the PEI adhesive—lead to fiber tear failures. Consequently, the optimal welding temperature for the skin and omega stiffener components was identified to be equal to 340 °C.

### 2.3. Press Consolidation Welding of Skin and Omega Stiffener

**Figure 5:**
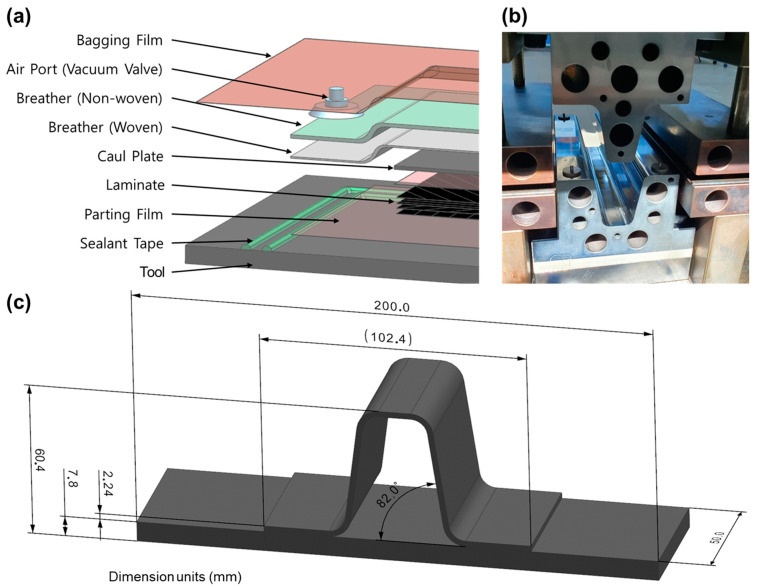
(**a**) Oven consolidation schematic of CF/PEKK thermoplastic composite skin. (**b**) Stiffener press-stamp thermoforming. (**c**) Dimensions of aircraft skin/stiffener specimen.

The manufacturing process of the skin and stiffener applied in the experiment and a drawing of the welded product are shown in Figure 5. The skin was consolidated using a vacuum bag oven process (Figure 5a), laminating a total of 56 plies in a quasi-isotropic layup sequence of [+45/0/−45/90]_7s_. The lamination process began by placing a parting film atop the tool, followed by the prepreg. Subsequently, a cowl plate, breather, and bagging film were layered in sequence, and a vacuum of 1 bar was applied. The entire parts were then oven-heated. The consolidation phase involved holding the sample at 380 °C for 1 h, followed by cooling to room temperature before ejection. Figure 5b shows the stamp forming tool for the stiffener’s forming tool applied in the experiment.

The stiffener was manufactured from a 16-ply laminate with the same [+45/0/−45/90]_2s_ configuration as the single-lap shear test specimen. The laminate was heated to 400 °C and then formed in a mold heated to 290 °C and ejected at 150 °C. The punch and die angles were modified to account for the spring-in that happens during the formation of the side wall and flange of the stiffener. Additionally, the punch and die were coated to ensure a smooth release. The coating was applied to prevent any defects that may arise on the welding surface due to wrinkles on the specimen’s surface caused by the use of a release film. Figure 5c shows a drawing of the final parts of the skin/stiffener assembly. The skin dimensions were designed to be 200 mm × 50 mm for pull-off tests. The stiffener had a side wall angle of 82°. The overlapping area of skin and stiffener was 25 mm × 50 mm.

**Figure 6:**
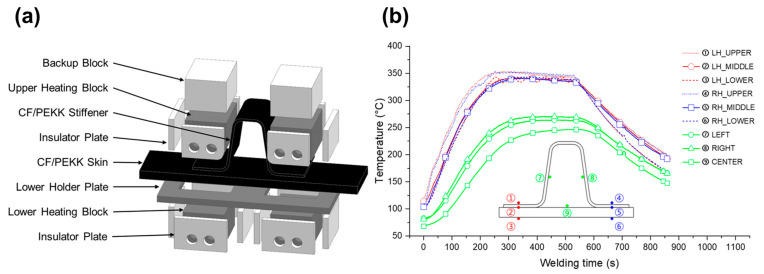
(**a**) Setup for press conduction welding of aircraft skin/omega stiffener specimen. (**b**) Thermal profile within the welding jig.

Figure 6 shows a drawing of the press conduction welding tool and data for evaluating the heating performance of the tool. Figure 6a presents a three-dimensional (3D) representation of the welding jig. To ensure temperature uniformity, a ceramic insulator was affixed to all sides except the contact area between the specimen and the heating block, and thus concentrated the heat within the overlapping flange region between the skin and omega stiffener. The alignment of the top and bottom heating blocks was achieved by using the base plate, while the demonstrator was accurately positioned for welding using a pilot pin on the lower holding plate. Cartridge heaters were employed to heat the block, with a PID method regulating the temperature to attain a target temperature of 340 °C at the interface between the skin and omega stiffener. Figure 6b depicts the thermal profile of the specimen mounted on the press conduction welding jig. To perform welding, the sample was positioned in a conduction welding device at a relatively low temperature. The sample was heated at the same time as the tool; therefore, the heating time varied based on the tool’s temperature due to the sample’s heating. To ensure steady heating, a technique was adopted to heat simultaneously the sample and the tool. Verification of unnecessary variables was performed based on temperature measurements to prevent the deformation of the specimen or joint area. The maximum temperature in areas not designated for welding reached 247 °C, and the omega stiffener’s left and right walls experienced marginally higher temperatures than the center of the skin. Consequently, to ensure consistent process conditions, the skin/omega stiffener specimen was maintained at an interface temperature of 340 °C and at a pressure of 1 MPa for a welding duration of 3 min. Three specimens were fabricated and tested.

## 3. Results

### 3.1. Pull-Off Test

**Figure 7:**
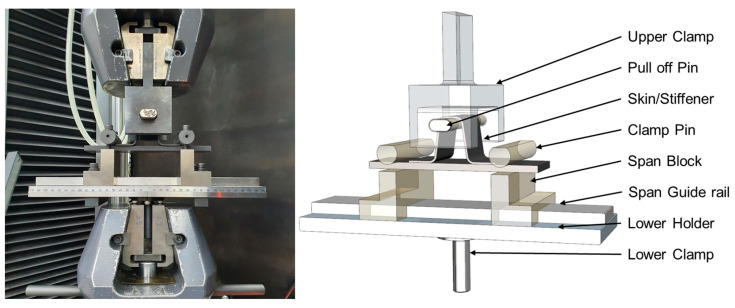
Fabrication and testing of pull-off test jig.

The bonding integrity of the skin/omega stiffener specimen was evaluated using compression and tensile testing. While compression testing may be preferred for assessing large-scale structures owing to its ability to simulate operational stress conditions, tensile testing is more commonly applied to smaller scale structures. To circumvent potential clamping issues during tensile testing—particularly those arising from drilling—a technique was adopted that tensioned the front of the stiffener’s upper surface. Typically, tensile tests are conducted by clamping the stiffener’s upper center with bolts. Consequently, a pull-off test jig was designed and fabricated to assess the specimen’s bonding performance effectively, as depicted in Figure 7. The test was performed by applying a tensile load to a pin inserted into the top of the omega stiffener.

**Figure 8:**
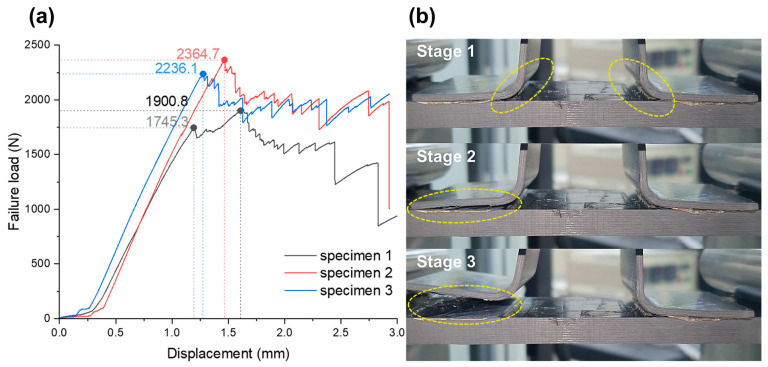
(**a**) Load–displacement graph for welding properties of skin/omega stiffener during pull-off test. (**b**) Pull-off test process of specimen 3.

The graph showing the relationship between the load and displacement followed a linear path until it reached the first peak. After this, there was a drop in the load, thus indicating an irregular deformation behavior. In Specimen 1, the maximum load occurred at approximately 0.5 mm after the decrease of the first load. For specimens 2 and 3, the first peak was the point of maximum load, and the fracture process took place in three stages, as shown in Figure 8b. At the initial peak, which occurred at the maximum load, debonding started at the corner of the omega stiffener flange (stage 1). As the cross-head moved, crack propagation became concentrated on one flange side (stage 2), until the reinforcing fibers bridged and finally detached (stage 3). Conversely, specimen 1 exhibited a gradual increase in load after the first peak and achieved maximum load at a displacement of 1.6 mm. Interestingly, during stage 2, specimen 1 exhibited crack propagation across both flanges, thus indicating that the occurrence of the first peak does not always align with the maximum load. After applying an even tensile load to all areas of the top with stiffeners, inconsistent results were produced, suggesting a complex failure mechanism with various variables in the experiment, possibly indicating a complex disintegration process.

It is possible that the deformation of the stiffener during the forming process could have caused geometric distortions. This could be why the load was not evenly transmitted to both flanges and, instead, concentrated on one flange, leading to the formation of cracks on one side. However, since the first peak is considered the most significant factor in evaluating performance, and due to the unique characteristics of composite materials, this phenomenon is not expected to have a high impact. From a product perspective, it is crucial to confirm the physical properties at the point of occurrence of the first peak, as it is a crucial factor in evaluating the performance of the product. It is possible that the uneven fracture of both flanges was caused by the influence of the squeezed PEI adhesive. The joint surface between the corner where the stiffener flange begins and the skin, has a shape that inevitably leads to stress concentration. To disperse the stress concentration in the corners and improve physical properties, most manufacturers use fillet (noodle) molding. However, in this experiment, there were limitations in controlling the area of squeezed resin, even though the PEI adhesives were arranged uniformly. Therefore, it is difficult to exclude the possibility that the irregular squeezing of the adhesive affected the stress transfer at the corner and acted as a variable in the formation of the first peak.

### 3.2. Fracture Surface Analysis

**Figure 9:**
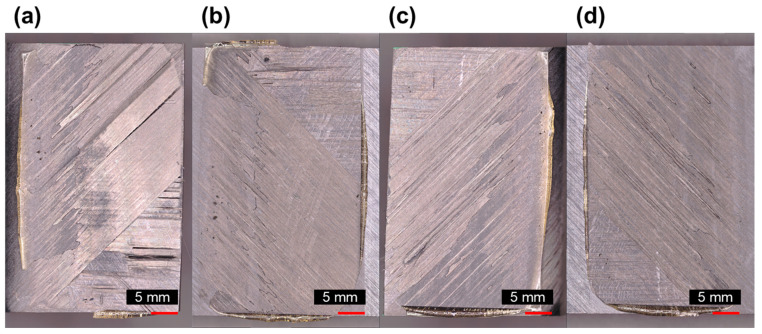
Photographs of fracture surface: (**a**) omega stiffener right flange, (**b**) right lap area of skin, (**c**) omega stiffener left flange, and (**d**) left lap area of skin.

**Figure 10:**
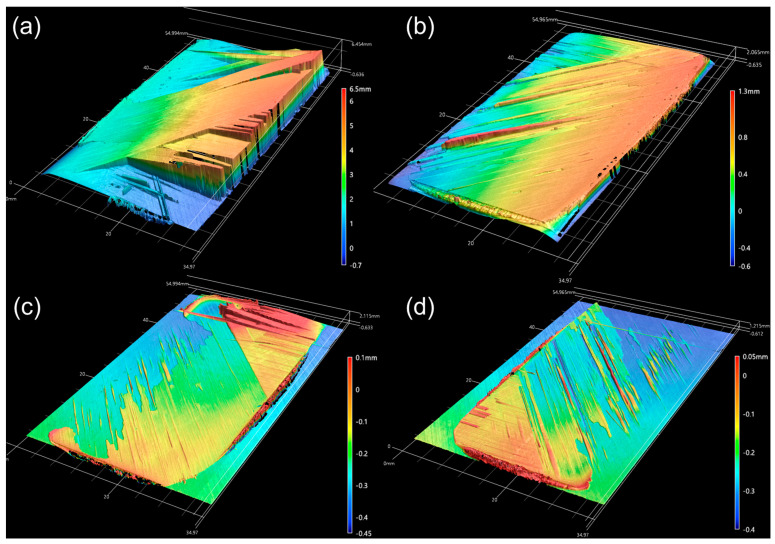
Three-dimensional noncontact measurements of the fractured surface: (**a**) omega stiffener right flange, (**b**) right lap area of skin, (**c**) omega stiffener left flange, and (**d**) left lap area of skin.

Microscopic examination of the fracture surface reveals critical insights into the failure mechanisms, as shown in Figure 9. The presence of resin, squeezed out by the PEI adhesive, was noted across the specimen’s entire surface area, with fiber tear failures occurring in specific zones. A significant observation is the predominance of interfacial failure at the 45° ply contacts rather than adhesive failure within the PEI welding layer, accompanied by delamination in the omega stiffener. Figure 9a,b illustrate the crack development and the bridging of reinforcing fibers in the interconnected region. Figure 9c,d shows fiber tear failure at the end region of one corner of the 45° ply. This can be more clearly observed in Figure 10, which presents the results of noncontact 3D measurements.

Figure 10 provides a detailed visualization of surface height variances through noncontact 3D measurements, thus enabling the observation of fiber tear failures in specific areas. Figure 10a highlights a crack at the interface that penetrates the laminate, thus causing delamination and exhibiting traces of bridged fiber pull-out. Bridged fibers (as small as two plies) were suspended in the air; accordingly, they cannot be easily distinguished with optical-based noncontact measurement equipment (see results in Figure 10a). Consequently, the shape protrudes approximately 6 mm from the interface, as depicted in Figure 10b. The adhesive residue’s surface is visible in the skin measurement results. Figure 10c,d reveals an interfacial failure of the 45° ply, albeit within a relatively narrow region. This deviation is attributed to the differential loads on the flange sides, as discussed in Section 3.1, where one side fractures under a higher load without simultaneous cracking on both sides. These results may occur due to clamping deviation errors during the pull-off test of the skin/stiffener assembly sample or warpage defect of stamp forming of the stiffeners. The findings suggest that delamination, as seen in Figure 10c,d, was facilitated by the squeezed-out lump of PEI adhesive, given the 45° ply’s susceptibility to delamination. Comparing these observations with the single-lap shear test results from Section 2.2, fiber tear failures were identified without accompanying fiber spreading and part deformation, thus indicating effective bonding. However, even though specimens were fabricated under uniform conditions, the emergence of several additional variables contributed to the irregular fracture type observed in the load-displacement graphs, ascribed to the propagation of cracks.

## 4. Discussion

Recent research on thermoplastic composite welding has mostly focused on joining processes that do not require adhesives. Most of these studies have looked at heating methods to solve problems such as fiber spreading and matrix degradation. However, this study deals with welding methods that use adhesives. When composite materials with high dimensional accuracy are molded, they may experience changes in their dimensions due to the melting of the matrix resin. Using adhesives can help prevent product deformations. Additionally, thermoplastic composites typically have lower resin content than thermoset composites, which can expose the reinforcement and become a concern. This paper discusses the benefits of using the press welding method, which conserves energy by heating and pressurizing the joint surface locally. This method is highly efficient because it produces a single product with just one operation of the press, enabling the use of faster, cheaper, and more reliable welding processes that result in excellent quality com-pared to other welding methods. However, more complex shapes may require a larger press or specialized tools, which can limit the size of the part that can be welded.

This study focused on the secondary bonding of a press-conduction welded aircraft skin/omega stiffener specimen constructed from CF/PEKK thermoplastic composite and film-type PEI adhesive. The process parameters were selected based on the results of single-lap shear tests, and the optimal conditions were subsequently applied to the press conduction welding of aircraft skin/omega stiffener specimens. The performance of the bonded parts was assessed through pull-off tests.

The bonding temperature was determined based on DSC measurements of the *T*_g_ of the PEI adhesive and *T*_m_ of the PEKK matrix to establish a process temperature range. The peak bonding strength observed at 340 °C was characterized by fiber tear failure, with surface fibers being pulled out and fractured. It is presumed that the squeezed PEI adhesive created a lump, causing the 45-degree fiber to break and promoting thickness direction breakage at the outer edge. Therefore, the fracture surface appeared to be a complex form of fiber tear failure and adhesive failure.

The pull-off test results for three aircraft skin/omega stiffener specimens, although produced under identical conditions, showed inconsistent trends. This inconsistency can be attributed to the influence of compressed adhesive that irregularly accelerated interfacial delamination at the edges of the welded region and facilitated crack occurrence at the interface, thus penetrating the laminate’s interior. These irregularities make it challenging to deduce consistent trends from the experimental results. In most cases, failure of the 45° layer was observed at the corner of the laminate, and sometimes the separated layer protruded from it. Fracture observation of the surface can be based on the influence of the previously described lumpy PEI adhesive through the bridging stiffener flange. The fracture is not clearly visible at the corner radius of the flange, where the load is first transmitted in contact with the skin.

However, at the end of the flange, torn fibers of the adherend can sometimes be observed, or the entire ply is torn, and bridging can be seen. Therefore, it can be inferred that the shape of the fracture surface may vary depending on the squeezed adhesive. It is possible that the PEKK matrix melted in a specific area during the heating process for welding, or it was caused by a structural issue in the adhesion between layers depending on the lay-up sequence. Thus, the fracture type was evaluated based on qualitative analysis of the fracture surface. Further research is needed to analyze and diversify the lamination angle of the joint surface in additional experiments, as well as to study the effect of a squeezed adhesive lump.

## 5. Conclusions

(1) The temperature range for the process was determined using DSC. The optimal conditions were then determined by performing a single-lap shear test. The results indicated that the highest bonding strength of approximately 19.5 MPa was achieved at a temperature of 340 °C. Conversely, no bonding was observed under conditions close to the crystallization temperature of PEKK. It is believed that this was due to insufficient melting of the PEI adhesive, which resulted in a lack of interdiffusion.
(2) Various types of composite failures, including fiber tear failure, were observed under the bonding conditions derived from the single-lap shear test, aligning with the findings from the pull-off tests of the skin/omega stiffener specimens.
(3) It is believed that the squeezed PEI adhesive lump causes the following effect. The formation of irregular lumps leads to the growth of cracks inside the laminate, which results in fiber tear failure and bridging of specific plies during the fracture process. As a result, it is assumed that there is a phenomenon where the maximum load and the first peak load do not match in the pull-off test results.
(4) The welding of thermoplastic composites typically excludes the use of adhesives owing to the reversible reaction characteristics of the matrix. However, this investigation aimed to analyze experimentally and highlight the challenges that must be addressed in the application of secondary bonding to thermoplastic composites. Based on this analysis, the study contributes to a deeper understanding of the mechanical behavior of bonded joints in thermoplastic composite structures and underscores the need for further research to refine bonding processes in aerospace applications. In order to minimize the influence of adhesive lump, it is necessary to conduct in-depth research on fatigue strength and physical properties after bonding through a peel strength-based double cantilever beam test and end-notch flexure test. Such research is being planned.

## Figures and Tables

**Table 1:** Single-lap shear test welding process parameter range.

Parameter	Details
Material	CF/PEKK [+45/0/−45/90]_2s_ 16 Ply, 2.24 mm
Adhesive	PEI film, 0.175 mm thickness
Welding pressure	1 MPa
Holding time	180 s
Welding temperature	260, 280, 300, 320, and 340 °C
Ejecting temperature	150 ± 10 °C
Cooling rate	0.6 °C/s

## Data Availability

The original contributions presented in the study are included in the article; further inquiries can be directed to the corresponding authors.
